# Supplementary material for: A Comparative Structure/Function Analysis of Two Type IV Pilin DNA Receptors Defines a Novel Mode of DNA Binding
Source: Structure. 2016 Jun 7;24(6):926–34. doi: 10.1016/j.str.2016.04.001 (PMC4906244; doi:10.1016/j.str.2016.04.001)
Supplement: Document S1. Figures S1–S3 and Tables S1 and S2 [file mmc1.pdf]

Structure, Volume *24*

Supplemental Information

A Comparative Structure/Function Analysis of Two Type IV Pilin DNA Receptors Defines a Novel Mode of DNA Binding

Jamie-Lee Berry, Yingqi Xu, Philip N. Ward, Susan M. Lea, Stephen J. Matthews, and Vladimir Pelicic

A comparative structure/function analysis of two type IV pilin DNA receptors defines a novel mode of DNA-binding

Jamie-Lee Berry[1], Yingqi Xu[2], Philip N. Ward[3], Susan M. Lea[3], Stephen J. Matthews[2,]* and Vladimir Pelicic[1,]*

[1]MRC Centre for Molecular Bacteriology and Infection, Imperial College London, London SW7 2AZ, United Kingdom

[2]Centre for Structural Biology, Imperial College London, London SW7 2AZ, United Kingdom

[3]Sir William Dunn School of Pathology, University of Oxford, Oxford OX1 3RE, United Kingdom

*Correspondence: s.j.matthews@imperial.ac.uk (S.J.M.), v.pelicic@imperial.ac.uk (V.P.)

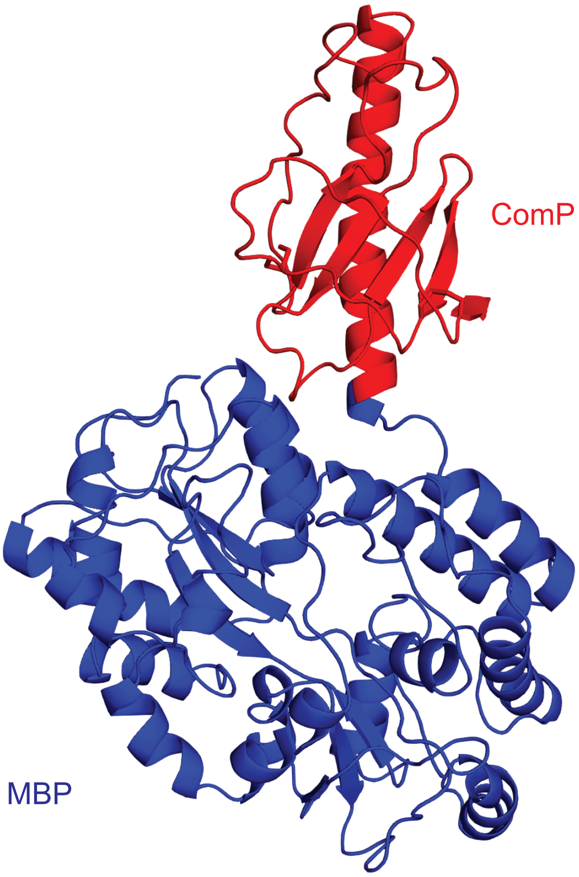

Figure S1, related to Figure 3. Full 3D structure of the MBP-ComP fusion protein. The crystal structure is shown as a cartoon drawing. The ComP moiety is highlighted in red, while the MBP moiety is highlighted in blue.

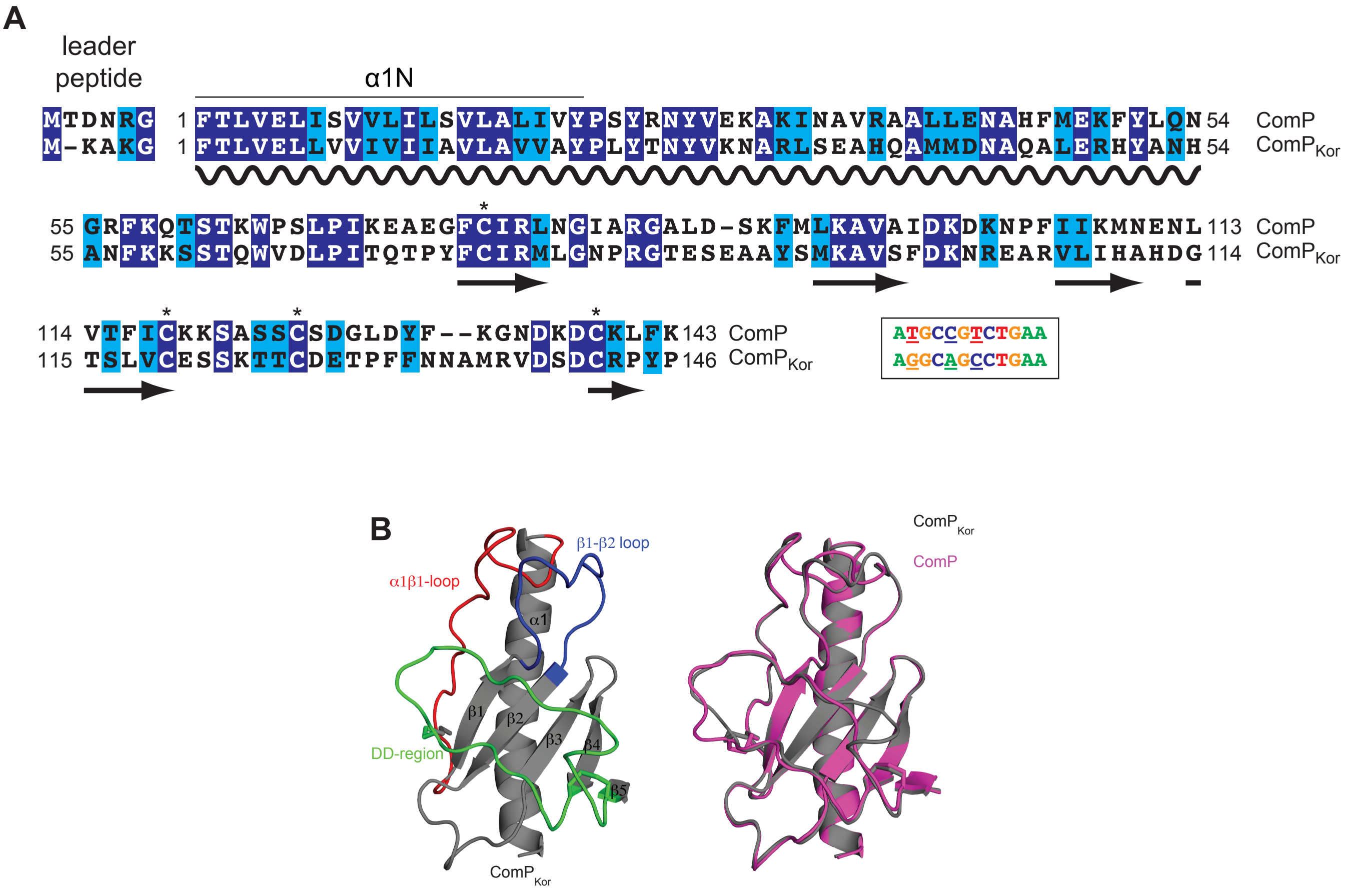

Figure S2, related to Figures 3 and 4. Homology modelling of the structure of the ComP$_{Kor}$ ortholog from *K. oralis*. (A) Sequence alignment of ComP$_{Kor}$ with ComP from *N. meningitidis* 8013, produced using Clustal Omega. Amino acids were shaded in dark blue (when identical), in light blue (when highly similar) or non-shaded (when non-conserved). Relevant structural and functional features have been highlighted. The four Cys residues that form two crucial disulfide bonds are identified by *. The inset represents a sequence alignment of DUS and the DUS variant found in *K. oralis*. These motifs differ by three base, which have been underlined. (B) Cartoon drawing representation of the homology model of ComP$_{Kor}$ and of its superposition with the ComP crystal structure.

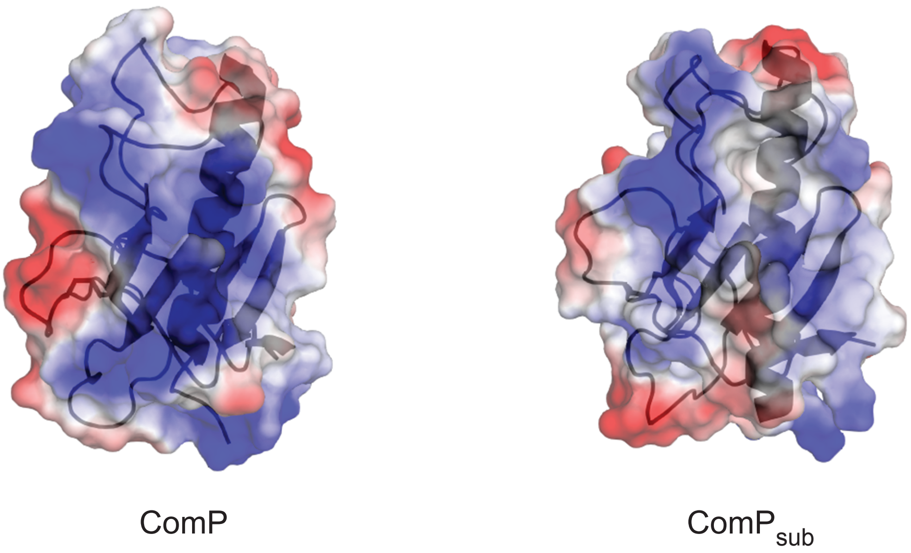

Figure S3, related to Figures 3 and 4. Surface charge representation of the ComP and ComP$_{sub}$ structures. Positively charged residues are represented in blue, negatively charged residues in red, while neutral residues are not coloured.

Table S1, related to Figures 3 and 4. Structural data collection and refinement parameters.

**MBP-ComP**	
Resolution range (Å)	58.05-1.43 (1.47-1.43)
Space group	$P2_12_12_1$
Unit cell dimensions	63.57, 68.44, 109.58; 90, 90, 90
Total observations	415,797 (28,880)
Total unique	88,446 (6,445)
Multiplicity	4.7 (4.5)
Completeness (%)	99.5 (99.2)
I/σ(I)	15 (1.2)
Wilson B factor	21.3
R_{merge}	0.039 (1.096)
R_{meas}	0.049 (1.384)
R_{work}	0.16
R_{free}	0.19
Ramachandran favoured (%)	97.2
Ramachandran outliers (%)	0
Average B factor	29
**His_6-$ComP_{sub}$**	
Number of distance restraints	2,017
intra-residual	744
sequential	445
medium range	273
long range	555
NOE violations >0.5 Å	1
Dihedral violations >5º	0
Ramachandran favoured (%)	75.5
Ramachandran allowed (%)	20.6
Ramachandran generously allowed (%)	3.9
Ramachandran disallowed (%)	0

Table S2, related to the Experimental proocedures. Primers used in this study.

Name	Sequence[a]
**Cloning**	
opt*comP*-F	gg**gaattc**GAAAAAGCCAAAATTAACGCAGTT
opt*comP*-R	gg**aagctt**TTATTTAAACAGTTTGCAGTCTTTG
comP$_{sub}$-pMalF	gg**gaattc**CGCTCGGCCAACCTGCGTG
comP$_{sub}$-pMalR	gg**aagctt**TCACCCCGTAAAAGGCCGA
his*comP*$_{sub}$-pETF	gg**ccatgg**atcatcatcatcatcatcatCGCTCGGCCAACCTGCGTG
his*comP*$_{sub}$-pETR	cc**ggatcc**TCACCCCGTAAAAGGCCGA
**DNA-binding assay (EMSA/SPR)**	
DUS$_{var1}$1[b]	tgaccAGGCCGTCTGAAcaaac
DUS$_{var1}$2	gtttgTTCAGACGGCCTggtca
SUD1[b]	tgaccACGACTTATAATcaaac
SUD2	gtttgATTATAAGTCGTggtca
SDU1[b]	tgaccAAGGCCTGTCATcaaac
SDU2	gtttgATGACAGGCCTTggtca
**NMR titration**	
DUS$_{var1}$1bis	cAGGCCGTCTGAAc
DUS$_{var1}$2bis	gTTCAGACGGCCTg

[a]Overhangs are in lower case, with restriction sites in bold.

[b]When indicated, these primers were 5'-labelled with biotin.
