# Supplementary material for: A Comparative Structure/Function Analysis of Two Type IV Pilin DNA Receptors Defines a Novel Mode of DNA Binding
Source: Structure. 2016 Jun 7;24(6):926–34. doi: 10.1016/j.str.2016.04.001 (PMC4906244; doi:10.1016/j.str.2016.04.001)
Supplement: Document S2. Article plus Supplemental Information [file mmc2.pdf]

Article

Structure

A Comparative Structure/Function Analysis of Two Type IV Pilin DNA Receptors Defines a Novel Mode of DNA Binding

Graphical Abstract

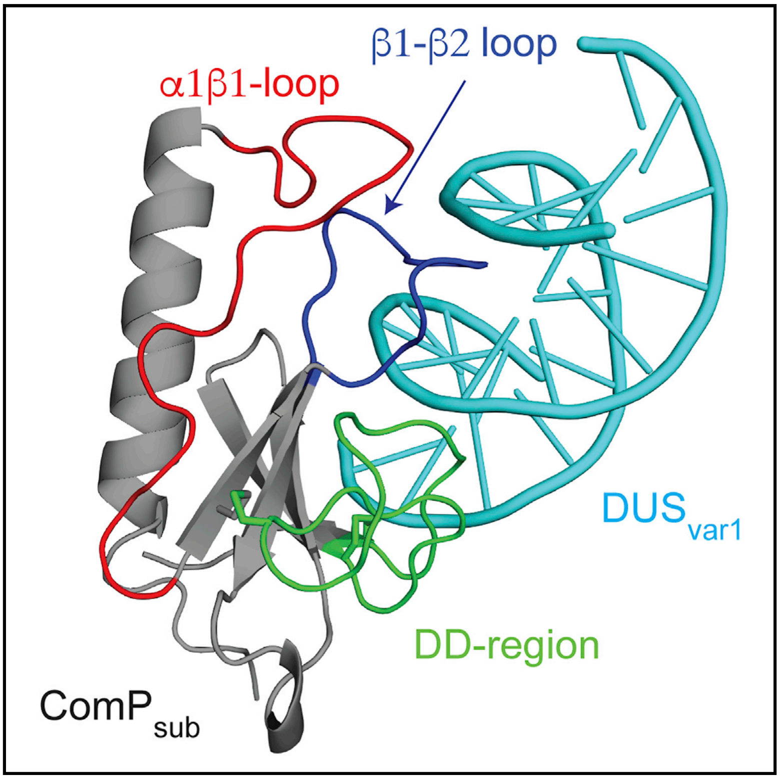

Authors

Jamie-Lee Berry, Yingqi Xu,
Philip N. Ward, Susan M. Lea,
Stephen J. Matthews, Vladimir Pelicic

Correspondence

s.j.matthews@imperial.ac.uk (S.J.M.),
v.pelicic@imperial.ac.uk (V.P.)

In Brief

ComP is the first type IV pilin with DNA-binding ability, key for natural transformation in Neisseriaceae. By reporting high-resolution structures for two ComP orthologs, Berry et al. shed light on a novel mode of DNA binding.

Highlights

- ComP orthologs are type IV pilins binding DNA, key for natural transformation
- Two high-resolution structures reveal a unique pilin fold
- DNA binding involves a novel motif

Accession Numbers

5HZ7
2NBA

Berry et al., 2016, Structure *24*, 926–934
June 7, 2016 ©2016 Elsevier Ltd All rights reserved
http://dx.doi.org/10.1016/j.str.2016.04.001

CellPress

Structure
Article

A Comparative Structure/Function Analysis of Two Type IV Pilin DNA Receptors Defines a Novel Mode of DNA Binding

Jamie-Lee Berry,[1] Yingqi Xu,[2] Philip N. Ward,[3] Susan M. Lea,[3] Stephen J. Matthews,[2,*] and Vladimir Pelicic[1,*]
[1]MRC Centre for Molecular Bacteriology and Infection, Imperial College London, London SW7 2AZ, UK
[2]Centre for Structural Biology, Imperial College London, London SW7 2AZ, UK
[3]Sir William Dunn School of Pathology, University of Oxford, Oxford OX1 3RE, UK
*Correspondence: s.j.matthews@imperial.ac.uk (S.J.M.), v.pelicic@imperial.ac.uk (V.P.)
http://dx.doi.org/10.1016/j.str.2016.04.001

SUMMARY

DNA transformation is a widespread process allowing bacteria to capture free DNA by using filamentous nano-machines composed of type IV pilins. These proteins can act as DNA receptors as demonstrated by the finding that *Neisseria meningitidis* ComP minor pilin has intrinsic DNA-binding ability. ComP binds DNA better when it contains the DNA-uptake sequence (DUS) motif abundant in this species genome, playing a role in its trademark ability to selectively take up its own DNA. Here, we report high-resolution structures for meningococcal ComP and *Neisseria subflava* ComP$_{sub}$, which recognize different DUS motifs. We show that they are structurally identical type IV pilins that pack readily into filament models and display a unique DD region delimited by two disulfide bonds. Functional analysis of ComP$_{sub}$ defines a new mode of DNA binding involving the DD region, adapted for exported DNA receptors.

INTRODUCTION

Numerous bacterial species, defined as naturally competent, are able to capture free DNA from the environment and import it into their cytoplasm across formidable permeability barriers (Chen and Dubnau, 2004). Although imported DNA can be used as a source of food or as a template for the repair of DNA damage (Chen and Dubnau, 2004), when the DNA is new and it is stably acquired, this process is called transformation, since the bacteria are "transformed" by exhibiting novel phenotypic traits. Transformation allows competent bacteria to evolve rapidly by promoting transfer of DNA between different species, an important evolutionary process known as horizontal gene transfer (HGT) (Thomas and Nielsen, 2005).

Most naturally competent species import extracellular DNA using the same two-step process (Chen and Dubnau, 2004). The first step involves DNA uptake mediated by type IV filamentous (Tff) nano-machines composed of type IV pilins (Berry and Pelicic, 2015), either long filaments known as type IV pili (Tfp) or elusive competence (pseudo)pili. It is widely accepted (Chen and Dubnau, 2004; Maier et al., 2004) that filaments bind free DNA and pull it across the outer membrane (in Gram-negative bacteria) and/or peptidoglycan (in Gram-positive species). This scenario is supported by the finding that in species where *bona fide* retractable Tfp are involved, as in the human pathogens *Neisseria meningitidis* and *Neisseria gonorrhoeae*, there is no DNA uptake when pilus retraction is abolished (Brown et al., 2010; Wolfgang et al., 1998). In the second step, once DNA is in the periplasm/pseudo-periplasm, it is bound by the ComE/ComEA receptor (Chen and Gotschlich, 2001; Provvedi and Dubnau, 1999; Seitz et al., 2014) and translocated across the cytoplasmic membrane by the Com machinery (Chen and Dubnau, 2004). Eventually, imported DNA is integrated in the chromosome in a RecA-dependent manner.

Until recently, no filament-localized DNA receptor had been identified and it was unknown how Tff nano-machines bound extracellular DNA. However, recent findings clearly showed that some type IV pilins can act as DNA receptors. We discovered that the minor Tfp component ComP, which is identical in meningococci and gonococci and is crucial for their competence (Brown et al., 2010; Wolfgang et al., 1999), is the only one of four *N. meningitidis* type IV pilins with intrinsic DNA-binding ability (Cehovin et al., 2013). Furthermore, quantitative DNA-binding experiments (Berry et al., 2013; Cehovin et al., 2013) showed that although purified ComP can interact with any DNA sequence, it displays a preference for the 12-bp DNA-uptake sequence (DUS) motif (Ambur et al., 2007). This motif is highly repeated in meningococcal and gonococcal genomes and enhances uptake by these species of DNA containing it (Goodman and Scocca, 1988). Selective uptake of their own DNA is a characteristic feature of Neisseriaceae (Frye et al., 2013) and Pasteurellaceae in which a different motif (termed USS) is found (Danner et al., 1980). This property is thought to protect these competent species from indiscriminate transformation by foreign DNA. Identification of ComP as the DUS receptor shed light on the long-standing mystery of how pathogenic *Neisseria* species manage to recognize and import their own DNA during transformation. The presence of ComP homologs in all other *Neisseria* species and most Neisseriaceae, some of which were shown to exhibit preferential uptake of their own DNA-containing DUS variants (Frye et al., 2013), suggests that co-evolution of DUS variants and cognate ComP receptors is an elegant mechanism for modulating HGT between competent species

This is an open access article under the CC BY license (http://creativecommons.org/licenses/by/4.0/).

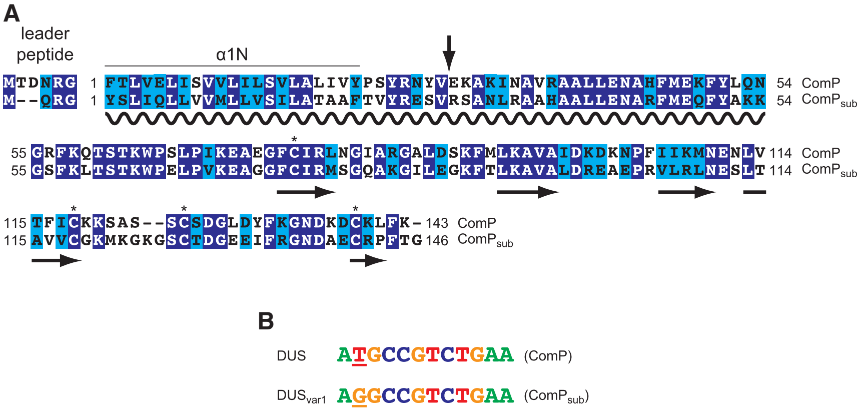

Figure 1. Comparison of ComP Orthologs in *N. meningitidis* and *N. subflava,* and of Their Cognate DUS Variants

(A) Sequence alignment of ComP and $ComP_{sub}$ from *N. meningitidis* 8013 and *N. subflava* NJ9703, produced using Clustal Omega. Amino acids are shaded in dark blue (when identical) or light blue (when highly similar), or non-shaded (when non-conserved). Relevant structural and functional features are highlighted. The proteins start with a conserved N-terminal sequence motif that defines all type IV pilins, the class III signal peptide (Szabó et al., 2007). This motif consists of a hydrophilic leader peptide, which is cleaved by the pre-pilin peptidase PilD, followed by a stretch of 21 predominantly hydrophobic residues that forms an extended α helix, which is the main assembly interface of subunits within filaments (Berry and Pelicic, 2015). To facilitate purification, we produced the recombinant proteins without their 28 N-terminal residues, depicted by an arrow. The four Cys residues that form two crucial disulfide bonds are identified by asterisks. The soluble portions that have been purified in this study, as well as the different structural motifs, are also highlighted.

(B) Sequence alignment of DUS and DUS_{var1} found in *N. meningitidis* and *N. subflava* genomes, respectively. These 12-bp motifs (Ambur et al., 2007) differ by just one base, which is underlined.

sharing the same environmental niche (Berry et al., 2013). This scenario is further supported by the finding that DUS variants present in other *Neisseria* species, which differ from meningococcal DUS by as little as 1 bp, show suboptimal transformation of *N. meningitidis* (Berry et al., 2013).

High-resolution structural information is necessary to advance our understanding of how the ComP family of type IV pilins recognizes DNA. Although a nuclear magnetic resonance (NMR) analysis generated a low-resolution global fold for meningococcal ComP and highlighted an electropositive surface important for DNA binding and transformation (Cehovin et al., 2013), the finer details were lacking. We therefore embarked upon a comparative structural analysis of two ComP orthologs. In the present study, we shine new light on this poorly understood and fascinating phenomenon by reporting high-resolution 3D structures for ComP from *N. meningitidis* and $ComP_{sub}$ from the non-pathogenic species *Neisseria subflava*, which is a common inhabitant of the human upper respiratory tract, and by performing an in-depth functional analysis of $ComP_{sub}$ DNA-binding ability, which shows specificity for DUS_{var1} differing from meningococcal DUS by 1 bp.

RESULTS

DNA Binding with a Preference for Their Cognate DUS Is a Conserved Property in ComP Homologs

Although purified $ComP_{sub}$ was previously shown to bind DNA (Berry et al., 2013), quantitative DNA-binding data were needed to strengthen the notion that all ComP homologs bind their cognate DUS specifically. To overcome previous protein stability problems, we fused the 118-amino-acid (aa) long soluble portion of $ComP_{sub}$ (Figure 1) to non-cleavable N-terminal maltose-binding protein (MBP) or hexahistidine tag (His_6). This allowed us to purify well-folded proteins, which we used to test whether $ComP_{sub}$ has a higher affinity for its cognate DUS_{var1}. First, we used acrylamide electrophoretic mobility shift assays (EMSA) to perform competition reactions. We assessed the effect of an excess of unlabeled double-stranded (ds) primer on a pre-formed complex between purified MBP-$ComP_{sub}$ and a biotinylated DUS_{var1} ds primer, which produces a characteristic shift on gel (Figure 2A). While unlabeled DUS_{var1} efficiently outcompeted bound biotinylated DUS_{var1} in a dose-dependent manner, as demonstrated by the gradual and eventually complete disappearance of the biotinylated complex, a scrambled SUD primer (in which every second base is altered) had no effect (Figure 2A). Next, the affinity of His_6-$ComP_{sub}$ for DUS_{var1} and two different scrambled primers was quantified in real time using surface plasmon resonance (SPR), as previously done for meningococcal ComP (Cehovin et al., 2013). In brief, equivalent amounts of ds biotinylated DUS_{var1}, SUD, and SDU primers were coupled to adjacent channels on a neutravidin-coated sensor chip. Increasing amounts of purified His_6-$ComP_{sub}$ (10, 25, 50, 100, or 200 μM) were then injected and the responses at equilibrium (R_{eq}) were measured for each protein concentration (Figure 2B). This clearly showed that $ComP_{sub}$ binds DNA in a dose-dependent fashion, and that the affinity for DUS_{var1} is much higher than the affinity for SUD or SDU, as indicated by the higher R_{eq} at each protein concentration. Unlike binding to the scrambled primer, binding to DUS_{var1} was approaching saturation, allowing us to estimate (using a non-linear regression least-squares fit) a dissociation constant (K_D) of 52.7 ± 2.2 μM. Taken together, these findings confirm that DNA binding, which is tighter in the presence of their cognate DUS, is a conserved property in ComP orthologs.

ComP Orthologs Share Similar 3D Structures with a Highly Distinctive DD Region Stabilized by Two Disulfide Bonds

Since there is no high-resolution structural data for this class of DNA-binding pilins, we first endeavored to solve the 3D structure of its defining member, *N. meningitidis* ComP. To facilitate purification and crystallization, we used a synthetic *comP* gene, codon-optimized for expression in *Escherichia coli*, and fused the 115-aa-long soluble portion of ComP to an MBP modified

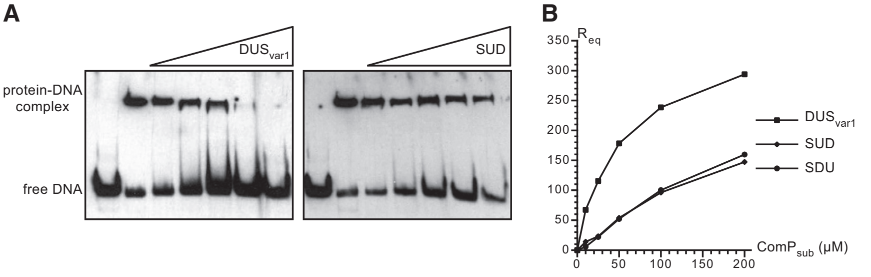

Figure 2. Quantitative Analysis of ComP$_{sub}$ DNA-Binding Propensity

(A) Analysis by competition EMSA. After pre-incubating biotinylated DUS$_{var1}$ ds primer with purified MBP-ComP$_{sub}$, increasing concentrations of unlabeled ds primers (DUS$_{var1}$ or SUD in which every second base was altered) were added to compete with bound DNA. DNA was then resolved by electrophoresis on native acrylamide gel, transferred to a positive nylon membrane, and detected using a streptavidin-horseradish peroxidase conjugate (Cehovin et al., 2013). In contrast to the DNA-only control (lane 1), a shift is seen in the presence of protein indicating the formation of an MBP-ComP$_{sub}$/DUS$_{var1}$ complex (lane 2). When the added unlabeled competitor DNA (lanes 3–7) displaces bound biotinylated DUS$_{var1}$, the shift disappears.

(B) Analysis by SPR. A neutravidin-coated sensor chip was used to immobilize (in different channels) similar amounts of biotinylated DUS$_{var1}$ and SUD ds primers, and increasing concentrations of purified His$_6$-ComP$_{sub}$ were injected. For each protein concentration, the responses at equilibrium (R_{eq}) were recorded. Results are the mean ± SD of four independent experiments.

to promote crystallization by surface entropy reduction (Moon et al., 2010). This soluble portion of ComP excludes the 6-aa-long leader peptide (Figure 1A), which is processed by the dedicated prepilin peptidase PilD (Brown et al., 2010), and the first 28 residues of the mature protein, most of which correspond to the hydrophobic residues that form the protruding part (α1N) of N-terminal α1 helix in type IV pilins (Berry and Pelicic, 2015). The MBP-ComP protein crystallized readily in multiple conditions. After optimizing the best diffracting crystals, we collected a complete dataset on crystals formed in the spacegroup $P2_12_12_1$, which diffracted to a resolution of 1.43 Å (Table S1), and solved the structure of the fusion protein (Figure S1). As can be seen in Figure 3, the ComP moiety adopts the classical type IV pilin fold (Giltner et al., 2012) with the C-terminal part of the long α1 helix (α1C) packed against a β sheet of four antiparallel β strands. This conserved core is highly similar to that of the major pilin PilE (Parge et al., 1995) and the minor pilin PilX (Helaine et al., 2007) (Figure 3B). Subtle differences in ComP's conserved core include the lack of curvature in α1C (as previously observed for PilX) and the rather long loop (14 residues) connecting β1 and β2, the first two β strands of the β sheet. In contrast, major differences exist in the structurally variable "edges" that usually distinguish type IV pilins (Giltner et al., 2012), the α1β1 loop connecting α1 and β1 and the C-terminal D region delimited by a disulfide bond (hence its name). These regions in ComP are unique (Figure 3). While ComP's α1β1 loop is an extended unstructured region, its D region is particularly striking. Unlike in (most) other type IV pilins, where one disulfide bond stabilizes the C terminus of the protein by stapling it to β4, in ComP the C terminus forms a long unstructured loop held in place across the face of the β sheet by two disulfide bonds between the four Cys residues in the protein (C_{76}-C_{125} and C_{118}-C_{139}). The C_{118}-C_{139} bond delimits the loop by stapling its C terminus back to β4 and is therefore equivalent to the single disulfide bond found in other pilins (Figure 3B). In contrast, C_{76}-C_{125} is unique and pins the middle of the loop back to β1. To highlight the unique nature of this region, we termed it the DD region. Finally, three of the last five residues of the protein form a short β strand (β5), which is incorporated into the β sheet (Figure 3A).

To determine the structural relationship between ComP orthologs that recognize different DUS, we also solved the structure of ComP$_{sub}$ from *N. subflava*. The 118-aa-long soluble portion of this protein, which has a binding preference for DUS$_{var1}$ (see Figure 2), displays ~52% sequence identity to ComP (Figure 1). Since we could not obtain crystals for the MBP-ComP$_{sub}$ fusion because the protein was too soluble, we decided to explore whether a solution structure determination by NMR would be possible for His$_6$-ComP$_{sub}$. We isotopically labeled our His$_6$-ComP$_{sub}$ with ^{13}C and ^{15}N for NMR assignment and obtained a high-resolution nuclear Overhauser effect (NOE)-derived structure in solution (Table S1). The ComP$_{sub}$ structures within the NMR ensemble superpose well onto each other, with a root-mean-square deviation (rmsd) of 0.28 Å for all backbone atoms, which suggests that there is no significant flexibility in the structure (Figure 4A). As can be seen in Figure 4B, ComP$_{sub}$ adopts a 3D structure highly similar to that of ComP. The two structures align over their whole length with an rmsd of 2.41 Å for all backbone atoms (Figure 4C). Importantly, the distinctive features highlighted in ComP are also present in ComP$_{sub}$, namely, the unstructured α1β1 loop, the long β1-β2 loop, and the DD region that is held in place across the face of the β sheet by two disulfide bonds between the four Cys residues (C_{76}-C_{127} and C_{118}-C_{141}) in ComP$_{sub}$.

Taken together, these structural findings show that ComP orthologs adopt very similar 3D structures and present a unique structural feature previously unreported in type IV pilins, i.e. a DD region that sits on top of the β sheet and is delimited by two disulfide bonds between four conserved Cys residues.

ComP Orthologs Bind DNA Mainly via Their DD Region and β1-β2 Loop, which Are Predicted to Be Exposed on the Surface of Tfp

As already mentioned, ComP's DNA-binding activity has been characterized to some extent by NMR (Cehovin et al., 2013). However, this analysis was limited by relatively poor NMR data with many key resonances broadened through conformational exchange, which rendered them unassignable and precluded the calculation of a high-resolution solution structure for ComP. In contrast, the NMR data for ComP$_{sub}$ is of much higher quality, leading to complete assignments of ^{1}H, ^{15}N, and ^{13}C nuclei and eventually to the calculation of a high-resolution solution structure (Figure 4). We therefore characterized by NMR the atomic resolution features of DNA binding by His$_6$-ComP$_{sub}$. We titrated increasing amounts of an unlabeled DUS$_{var1}$ ds primer

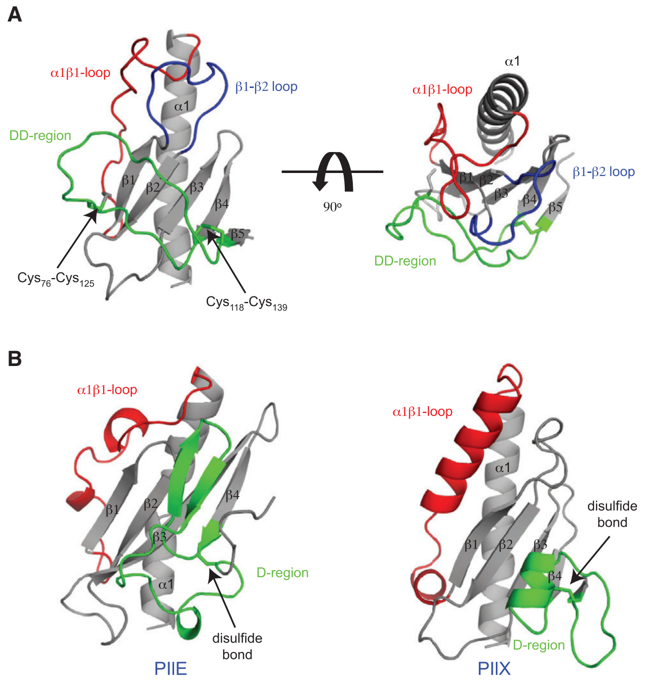

Figure 3. High-Resolution 3D Structure of the DUS Receptor ComP from *N. meningitidis*

(A) Crystal structure of the soluble portion of ComP at 1.43-Å resolution. Two different views are shown as a cartoon. The conserved core in type IV pilins (the N-terminal α helix and four-stranded antiparallel β sheet) is depicted in gray. Distinctive/key structural features such as the α1β1 loop (red), the large β1-β2 loop (blue), and the DD region delimited by two disulfide bonds that sits on top of the β sheet (green) are also highlighted.

(B) Structural similarity/differences between the soluble portions of ComP, major pilin PilE from *N. gonorrhoeae* (Parge et al., 1995), and minor pilin PilX from *N. meningitidis* (Helaine et al., 2007). The distinctive/key structural features shown in (A) are also highlighted using the same coloring scheme.

into isotopically labeled His$_6$-ComP$_{sub}$ and monitored chemical-shift perturbations (CSP). This analysis revealed significant and specific CSP (Figure 5A), which can be attributed to the interaction between the two molecules. Detailed inspection of $^1H^{15}N$ heteronuclear single-quantum coherence (HSQC) spectra revealed that the ComP$_{sub}$ residues undergoing CSP in the presence of DUS$_{var1}$ coalesce into three main contiguous "patches" (Figure 5B). The first patch corresponds to the large β1-β2 loop together with β2, the second patch corresponds to the first half of the DD region, while the last patch corresponds to the second half of the DD region. A small part of the α1β1 loop is also involved. Strikingly, these regions of ComP$_{sub}$ form an almost vertical "stack" along one face of the protein (Figure 5C). The residues experiencing CSP with low concentrations of DNA, and are thus likely to contact DNA first, lie almost exclusively in the DD region and the large β1-β2 loop (Figure 5). The residues experiencing CSP at higher concentrations of DNA are part of the β sheet, which is initially covered by the DD region and β1-β2 loop in the 3D structure. This suggests that there might be conformational changes in the protein upon DNA binding, leading to multiple binding modes.

The interaction between ComP$_{sub}$ and DUS$_{var1}$ led to the broadening of many signals even under saturating DNA concentrations, suggesting that even in the bound state there are significant conformational dynamics. Since this precluded the direct measurement of intermolecular NOEs, we used the HADDOCK software suite (de Vries et al., 2007; Dominguez et al., 2003) to generate a structural model for the ComP$_{sub}$-DUS$_{var1}$ complex. All the bases of DUS$_{var1}$ were considered as important, because the mutagenesis of even a single base significantly impairs DNA binding and/or transformation (Berry et al., 2013; Frye et al., 2013). In contrast, active residues in ComP$_{sub}$ were defined as those experiencing CSP at DNA concentrations below 20 μM (1/5 ratio of DNA to protein), to avoid giving weight to residues showing CSP at later titration points. The lowest overall energy cluster contained ten structures with an rmsd of 2.5 Å and an overall HADDOCK score of 70.7 ± 14.5 (Figure 6A). Complex formation in this cluster resulted in an average buried surface area of 1,558.1 ± 137.3 Å^2. Closer inspection of this complex reveals that the DNA docks onto the vertical stack of residues on ComP$_{sub}$ just above the ledge feature introduced by the DD region. The DD region, β1-β2 loop, and α1-β1 loop intercalate with successive grooves of the DNA, establishing contacts with multiple bases of DUS$_{var1}$ (Figure 6A). Finally, using Modeller (Webb and Sali, 2014), we produced a full-length ComP$_{sub}$ model using the gonococcal PilE structure (Craig et al., 2006) as a template for the missing N-terminal α1N helix and were able to model its packing within a Tfp structural model (Craig et al., 2006). This revealed that ComP$_{sub}$ not only fits readily into the filaments, but that the regions of the protein involved in DNA binding are clearly exposed on the surface of Tfp (Figure 6B).

Taken together, these findings suggest that ComP$_{sub}$ binds DNA using unique structural features conserved in this class of type IV pilins and exposed on the surface of the Tfp. These features, i.e. the large β1-β2 loop and DD region, define a new DNA-binding motif differing dramatically from well-known motifs (Luscombe et al., 2000).

DISCUSSION

Natural transformation is a widespread biological property playing a key role in bacterial physiology. Although it has been the subject of intense study for almost a century (Griffith, 1928) its very first step, during which Tff nano-machines bind free DNA to subsequently promote its uptake, remains one of the least understood. The discovery that ComP is the only purified meningococcal pilin capable of binding DNA showed that type IV pilins (the Tff subunits) can act as DNA receptors (Cehovin et al., 2013), highlighting a new property for these versatile molecular modules (Giltner et al., 2012). Furthermore, the finding that ComP shows better binding to the 12-bp DUS sequence motif (Ambur et al., 2007), which is highly abundant in pathogenic *Neisseria* species genomes and enhances uptake of their own DNA (Goodman and Scocca, 1988), solved a long-standing mystery. It revealed an elegant mechanism for limiting transformation by foreign DNA that is widespread in competent Neisseriaceae (Frye et al., 2013). A significant barrier to our understanding of how and why the ComP family can bind DNA is the absence of high-resolution structural information for this class of type IV pilins. In this report, we have addressed this limitation, which led to significant findings.

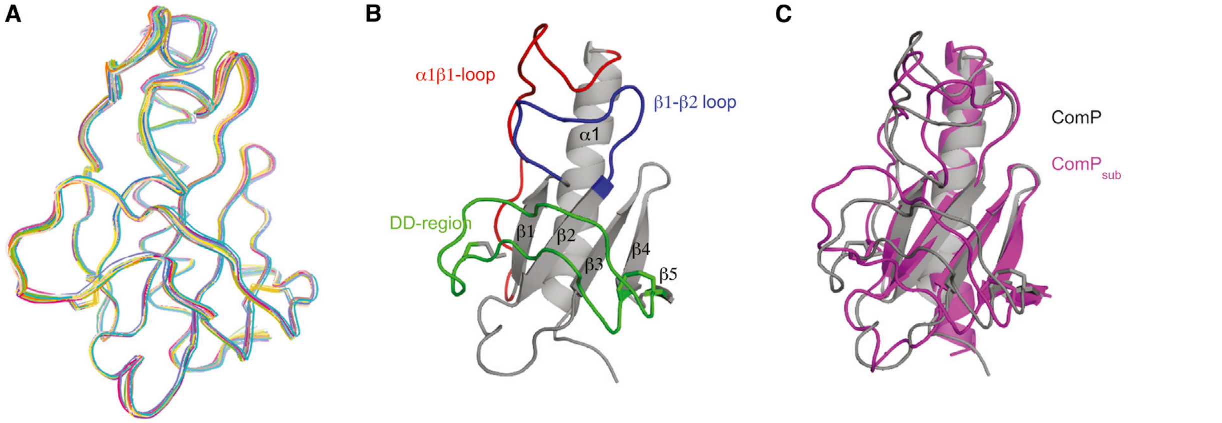

Figure 4. High-Resolution 3D Structure of the DUS$_{var1}$-Recognizing ComP$_{sub}$

(A) Ribbon representation of the superposition of the ensemble of 20 ComP$_{sub}$ structures determined by NMR.

(B) Cartoon representation of the ComP$_{sub}$ structure. The conserved core in type IV pilins (the N-terminal α helix and four-stranded antiparallel β sheet) is depicted in gray. Distinctive/key structural features such as the α1β1 loop (red), the large β1-β2 loop (blue), and the DD region delimited by two disulfide bonds that sits on top of the β sheet (green) are also highlighted.

(C) Cartoon representation of the superposition of ComP$_{sub}$ (magenta) and ComP (gray) structures. The two structures superpose with an rmsd of 2.41 Å over their entire length.

We first provide evidence suggesting that all ComPs are DNA-binding pilins displaying binding preference for their cognate DUS. Our previous finding that meningococcal ComP has a higher affinity for its cognate DUS (Cehovin et al., 2013) could readily be extended to other *Neisseria* species that contain the same DUS and encode ComPs with ~80%–100% aa identity (such as *N. gonorrhoeae*, *Neisseria lactamica*, *Neisseria polysaccharea*, and *Neisseria cinerea*). However, to generalize our findings to this entire class of proteins, it was still to be demonstrated that a ComP from more distant Neisseriaceae, which usually display ~30%–50% aa identity to meningococcal ComP (Cehovin et al., 2013), would show a similar binding preference to its cognate DUS that differs from meningococcal DUS by one to four bases (Frye et al., 2013). A previous competition EMSA with purified ComP$_{sub}$ fell short of this goal, although it showed a slightly better competition by its cognate DUS$_{var1}$ (Berry et al., 2013). No quantitative DNA-binding data could be obtained because of protein stability/folding issues. These problems were solved here by using novel expression/purification strategies. Using SPR, the affinity of ComP$_{sub}$ for its cognate DUS$_{var1}$ was found to be much higher than its affinity for scrambled primers. The K_D of ComP$_{sub}$ for DUS$_{var1}$ (53 μM) was found to be comparable with the K_D of meningococcal ComP for its cognate DUS (29 μM) measured previously (Cehovin et al., 2013). However, it is worth noting that these affinities, which are significantly lower than those for "classical" sequence-specific DNA-binding proteins such as transcription factors, might be underestimated due to the use of purified proteins. Indeed, it is not unlikely that ComP's affinity for DNA is higher when this protein is in its natural location within a Tfp, and would be further increased by the expected incorporation of multiple ComP subunits within a single filament. Together, these could cooperate to recognize one target DNA molecule with very high affinity and specificity.

We have also derived high-resolution structures for ComP and ComP$_{sub}$ orthologs. Consistent with the notion that they are minor components of Tfp (Aas et al., 2002; Brown et al., 2010), these structures revealed that ComP and ComP$_{sub}$ are *bona fide* type IV pilins and can pack efficiently within the available model for Tfp (Craig et al., 2006). Interestingly, these proteins also exhibit a distinctive structural feature exposed on the surface of the pilus, the DD region delimited by two disulfide bonds, which sits across the β sheet. The fact that this structural motif is not found in the dozens of type IV pilin structures available in the databases (Giltner et al., 2012) is consistent with ComP orthologs being (so far) the only known type IV pilins that bind DNA. Considering that (1) the 3D structures of ComP and ComP$_{sub}$ that share 50% of their residues are highly similar, (2) there is significant sequence homology even with more distant orthologs (~30% aa are identical) (Cehovin et al., 2013), and (3) all the DUS motifs share a conserved core, it is likely that all ComP orthologs will display the same 3D structure. This assumption is supported by homology modeling (using Modeller and ComP as a template) of the more distant ComP$_{Kor}$ ortholog from *Kingella oralis*, which shares only 27% aa identity with ComP and is expected to recognize a DUS differing from meningococcal DUS by three bases (Figure S2A). ComP$_{Kor}$ structural model was found to be virtually identical to meningococcal ComP structure since they align over their whole length with an rmsd of 0.26 Å for all backbone atoms (Figure S2B). Once other DNA-binding pilins are identified (that are not homologous to ComP), it will be interesting to explore whether they share a similar structure to ComP and whether the DD region is a conserved DNA-binding motif. This will be particularly interesting once the USS receptor in Pasteurellaceae is identified, because no ComP homolog is present in these species and their USS bears no resemblance to DUS motifs.

The last, and perhaps most important, finding in this study is that the mode whereby ComP orthologs interact with DNA has not been seen before. Rather than well-known and widespread DNA-binding motifs (helix-turn-helix, zinc finger, leucine zipper, and so forth) (Luscombe et al., 2000), ComP orthologs exploit a series of residues in two distinct domains, i.e. the DD region

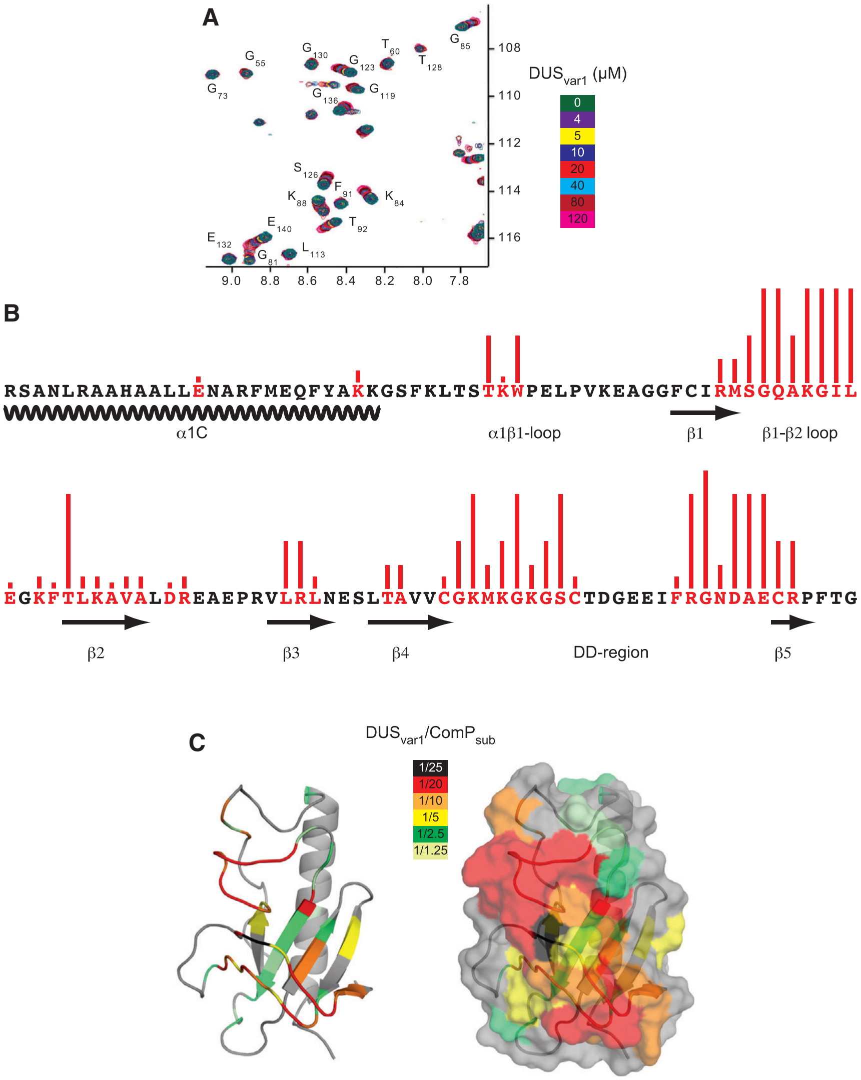

Figure 5. Detailed NMR Analysis of ComP$_{sub}$ Binding to DNA

(A) Overlay of representative portions of $^{1}H^{15}N$-HSQC NMR spectra for free His$_6$-ComP$_{sub}$ and His$_6$-ComP$_{sub}$ titrated with increasing concentrations of DUS$_{var1}$ ds primer. Labels are placed close to the peaks in the free state.

(B) DUS$_{var1}$-induced chemical-shift perturbations (CSP) mapped on the sequence of ComP$_{sub}$. The residues affected by DUS$_{var1}$ titration are colored red. The height of the bars above the affected residues is inversely proportional to the concentration of DNA at which CSP were detected.

(C) DUS$_{var1}$-induced CSP mapped on the 3D structure of ComP$_{sub}$ in cartoon and surface representations. The residues affected by DUS$_{var1}$ titration are gradient-colored according to the concentration of DNA needed, from lowest (black) to highest (light green).

and the tip of the β1-β2 loop (although a small part of the α1-β1 loop is also involved), to establish contacts with multiple bases of DUS in successive grooves of the ds DNA. These residues form an almost vertical stack on a face of the protein predicted to be exposed on the surface of the filaments, which was previously implicated in meningococcal ComP binding to DUS (Cehovin et al., 2013). As previously noted, this surface is highly positively charged (Figure S3) and is likely to be involved initially in electrostatic attraction of the negatively charged DNA. Interestingly, the DD region and the β1-β2 loop, which are flanked by almost invariable residues, are among those regions that differ the most between ComP orthologs (see Figures 1 and S2). This is likely to be the reason why these proteins recognize different DUS motifs. It is now possible to propose a model for the mode of action of these DNA receptors in natural transformation (Figure 7). When Neisseriaceae encounter free DNA they might attract it, electrostatically at first, and use ComP subunits in their Tfp to "scan" it for the presence of their cognate DUS. Once a cognate DUS has been recognized (it is unknown at this stage which specific aa differences in different ComPs are responsible for their different DUS specificities), ComP "docks" onto it using the vertical stack residues exposed on the filament surface, which is probably accompanied by conformational changes further increasing the strength and specificity of the interaction. Upon rotation of the pilus during PilT-powered retraction (Nivaskumar et al., 2014), the DNA docked at ComP anchor point(s) might wrap around the Tfp following the previously noted grooves on their corrugated surface (Craig et al., 2006). Eventually, upon translocation across the secretin pore, this would result in DNA uptake.

In conclusion, by providing high-resolution structural information for the ComP class of proteins, this study has shed light on the atomic basis for their DNA-binding ability, which is yet an additional property for the highly versatile type IV pilins. This has led to the discovery of a novel DNA-binding domain, which is consistent with the fact that members of the ComP family are, to the best of our knowledge, the only known surface-exposed DNA receptors that bind specific DNA sequences.

EXPERIMENTAL PROCEDURES

Protein Production and Purification of *N. meningitidis* ComP

A synthetic gene, codon-optimized for *E. coli* expression, encoding ComP from *N. meningitidis* 8013 (Rusniok et al., 2009), was synthesized by GeneArt. The portion of optimized *comP* encoding residues 29–143 from the mature protein was PCR amplified using opt*comP*-F and opt*comP*-R primers (Table S2), cut with *Eco*RI and *Hin*dIII and cloned into the pMALX(E) vector cut with the same enzymes. This resulted in the fusion of the soluble portion of ComP with a modified MBP carrier containing several mutations designed to promote crystallization (Moon et al., 2010). The resulting plasmid was verified by sequencing and transformed into chemically competent *E. coli* SHuffle T7 express cells (New England Biolabs), which enables formation of disulfide bonds in the cytoplasm. A single colony was transferred to 5 ml of Luria-Bertani (LB) medium (Difco) supplemented with ampicillin (100 μg ml^{-1}) and allowed to grow to saturation at 30°C overnight in an orbital shaker. The

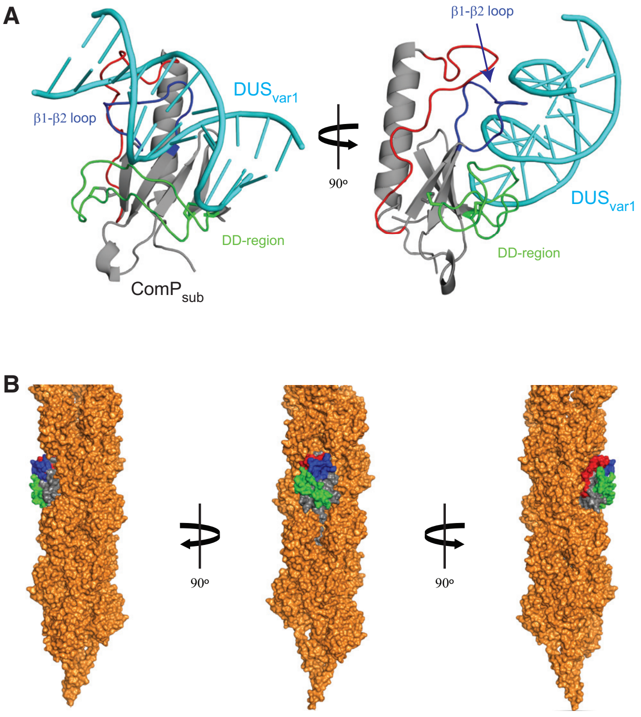

Figure 6. Modeling of the ComP$_{sub}$-DUS$_{var1}$ Complex and ComP$_{sub}$ Packing within Tfp

(A) HADDOCK model of the interaction between ComP$_{sub}$ (in which the α1β1 loop, β1-β2 loop, and DD region are highlighted) and DUS$_{var1}$ (in cyan) in cartoon representation. Two different views are shown.

(B) Packing of full-length ComP$_{sub}$ into Tfp. A full-length ComP$_{sub}$ model was generated using Modeller. One PilE subunit in the Tfp model was then replaced by this full-length ComP$_{sub}$. The structural features of ComP$_{sub}$ involved in DNA binding are highlighted on the surface representation.

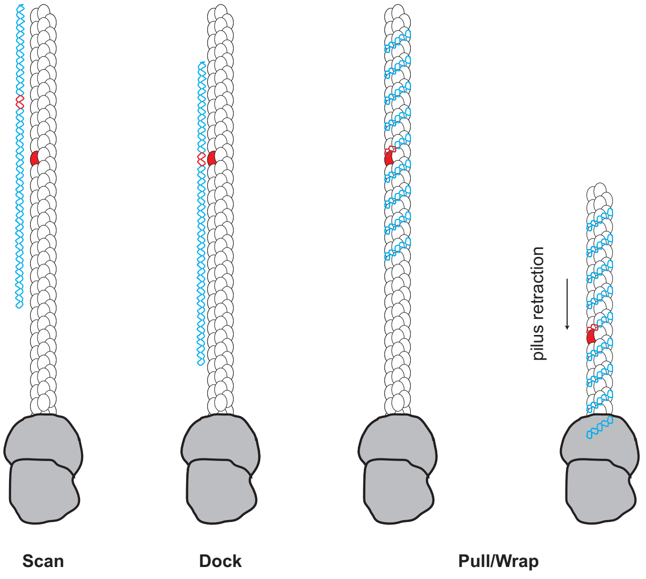

Figure 7. Model for the Role of ComP DNA-Binding Pilins in Natural Transformation in Neisseriaceae

After ComP (red pilus subunit) "scans" the DNA (cyan) for its cognate DUS (red), it "docks" onto this motif promoting tight binding, which allows DNA to be "pulled and wrapped" upon pilus retraction.

following day, this pre-culture was then used to inoculate 1 l of fresh LB-ampicillin and grown at 30°C in an orbital shaker until the OD_{600} reached 0.6–0.8. The culture was then placed into an orbital shaker set at 16°C and allowed to cool for 30 min, before adding 0.4 mM isopropyl β-D-1-thiogalactopyranoside (IPTG; Merck Chemicals) to induce protein production during 16 hr. Cells were then harvested by centrifugation at 8,000 × *g* for 20 min and subjected to one freeze/thaw cycle in lysis buffer A (50 mM Tris-Cl [pH 8], 100 mM NaCl, 1× SIGMAFAST EDTA-free protease inhibitor cocktail [Sigma]). This lysate was further disrupted by repeated cycles of sonication, i.e. pulses of 5 s on and 5 s off during 3–5 min, until the cell suspension was visibly less viscous. The cell lysate was then centrifuged for 30 min at 18,000 × *g* to remove cell debris. The clarified lysate was then passed using an Akta Purifier FPLC through a 5-ml MBPTrap HP column (both from GE Healthcare), pre-equilibrated in lysis buffer A, to bind MBP-ComP. The column was then washed extensively with lysis buffer A to remove unbound material before the fusion protein was eluted using TSM buffer (50 mM Tris-Cl [pH 8], 100 mM NaCl, 10 mM maltose). The affinity-purified MBP-ComP was further purified by gel-filtration chromatography on a HiLoad 16/60 Superdex 200 column using TSM buffer for elution.

Protein Production and Purification of *N. subflava* ComP$_{sub}$

For assessment of the DNA-binding activity of ComP$_{sub}$ by EMSA, the portion of *N. subflava comP* encoding residues 29–146 from the mature ComP$_{sub}$ protein was PCR amplified from *N. subflava* NJ9703 genomic DNA (Marri et al., 2010) using *comP*$_{sub}$-pMalF and *comP*$_{sub}$-pMalR primers (Table S2) and cloned as above into the pMALX(E) vector. This resulted in the fusion of the soluble portion of ComP$_{sub}$ with a non-cleavable MBP carrier. The fusion MBP-ComP$_{sub}$ protein was purified similarly to MBP-ComP.

For determination of the 3D structure of ComP$_{sub}$, the above portion of *N. subflava comP* was amplified using his*comP*$_{sub}$-pETF and his*comP*$_{sub}$-pETR primers (Table S2) and cloned into the pET-28b vector (Novagen). The forward primer was designed to fuse a non-cleavable N-terminal His_6 tag to ComP$_{sub}$. The resulting plasmid was verified by sequencing and transformed into chemically competent *E. coli* SHuffle T7 express cells. A single colony was transferred to 2 ml of LB medium supplemented with 50 μg ml^{-1} kanamycin and grown at 30°C to an OD_{600} of ~0.5. This pre-culture was back-diluted 1:50 into 10 ml of M9 minimal medium supplemented with a mixture of vitamins and trace elements. This was grown to saturation overnight at 30°C in an orbital shaker, then back-diluted 1:500 into 1 l of the same medium containing ^{13}C D-glucose and ^{15}N NH_4Cl for isotopic labeling. Cells were grown in an orbital shaker at 30°C until the OD_{600} reached 0.8, before adding 0.4 mM IPTG to induce protein production overnight at 30°C. Cells were then harvested and disrupted as above in lysis buffer B (50 mM Na_2HPO_4/NaH_2PO_4 [pH 7.4], 500 mM NaCl, 20 mM imidazole, 1× SIGMAFAST EDTA-free protease inhibitor cocktail). 2 ml of Ni-NTA (nitrilotriacetic acid) agarose resin (Qiagen), pre-washed in lysis buffer B, was then added to the clarified lysate and incubated for 2 hr at 4°C with gentle agitation. This chromatography mixture was then filtered through a Poly-Prep gravity-flow column (Bio-Rad) and washed several times with lysis buffer B, before eluting the protein with lysis buffer B containing 500 mM imidazole. The affinity-purified His_6-ComP$_{sub}$ was further purified by gel-filtration chromatography on an Akta Purifier using a Superdex 75 10/300 Gl column (GE Healthcare), and simultaneously buffer-exchanged into 50 mM Na_2HPO_4/NaH_2PO_4 (pH 6) and 50 mM NaCl.

Crystallization and Structure Determination of Meningococcal ComP

Purified MBP-ComP was concentrated to 7, 14, and 28 mg ml^{-1} and placed through sitting-drop vapor diffusion crystallization trials in MRC two-well crystallization plates (Hampton Research) using a wide range of commercially available kits. Trials were initially conducted using 100 nl of protein and 100 nl of mother liquor, over a reservoir of 80 μl of mother liquor. The trials produced a large number of initial hits, which were varied and optimized to yield larger and better diffracting crystals by altering the pH, precipitant concentration, and drop size. The crystals used for structure determination were obtained when the purified protein at 14 mg ml^{-1} was mixed 1:1 with crystallization liquor

containing 0.1 M sodium cacodylate (pH 6.5) and 25% polyethylene glycol 4000, and left to equilibrate with the reservoir solution at 20°C. Crystals were cryoprotected using crystallization liquor containing 20% ethylene glycol and flash-cooled in liquid nitrogen. Data were collected on beamline I02 at the Diamond Light Source and processed with xia2 (Winter, 2010). Molecular replacement was carried out using Phaser MR (McCoy et al., 2007) with MBP structure (PDB: 1HSJ) as a search model. ComP was then built manually into the remaining density using Buccaneer (Cowtan, 2006) and Coot (Emsley et al., 2010). Multiple iterative rounds of refinement and model building were carried out using Phenix (Adams et al., 2010) and Coot, resulting in a final R_{work}/R_{free} of 0.16/0.19.

NMR Structure Determination of $ComP_{sub}$

Isotopically labeled purified His_6-$ComP_{sub}$ was concentrated to ~750 μM in NMR buffer (50 mM Na_2HPO_4/NaH_2PO_4 [pH 6], 50 mM NaCl, 10% D_2O). A full set of triple-resonance NMR spectra was recorded on a Bruker Avance III 800-MHz spectrometer equipped with triple-resonance cryoprobes at 295 K, and processed with NMRPipe (Delaglio et al., 1995). Backbone assignments were completed using a combination of HBHA, HNCACB, HNCO, HN(CA)CO, and CBCA(CO)NH experiments using NMRView (One Moon Scientific) as previously described (Johnson and Blevins, 1994). Side-chain resonance assignments were obtained from a combination of CC(CO)NH, HC(C)H-total correlation spectroscopy (TOCSY), and (H)CCH-TOCSY experiments using an in-house software developed within NMRView (Marchant et al., 2008). Distance restraints were obtained from 3D $^1H^1H^{15}N$-NOE and $^1H^1H^{13}C$-NOE spectra and used for structure calculations in ARIA 2.3 (Rieping et al., 2007), along with dihedral angle restraints obtained from chemical-shift values calculated using the TALOS+ server (Shen et al., 2009). During later rounds of calculations, disulfide restraints for C_{76}-C_{127} and C_{118}-C_{141} were introduced, after these had already formed in the calculated structures. For each round of calculations, 20 structures were calculated over eight iterations. In the final iteration, the 20 lowest-energy structures were submitted to a water-refinement stage to form the final structural ensemble.

NMR Chemical-Shift Perturbation Analysis in $ComP_{sub}$ upon DNA Binding

The residues in $ComP_{sub}$ important for DNA binding were identified by NMR. Complementary primers corresponding to DUS_{var1} were first dissolved in NMR buffer to a final concentration of 6 mM, combined in equimolar amounts, heated for 5 min at 95°C and left to cool overnight to produce 3 mM ds target DNA. The ds primer was washed with NMR buffer using G-25 spin columns (GE Healthcare) to remove residual buffer components from the oligonucleotide synthesis. The ds target DNA was titrated into a 100 μM sample of His_6-$ComP_{sub}$ in NMR buffer, at increasing concentrations (4, 5, 10, 20, 40, 80, and 120 μM), and 2D $^1H^{15}N$-HSQC spectra were recorded at each titration point revealing changes to backbone chemical shifts.

DNA-Binding Assays

DNA binding by $ComP_{sub}$ was assessed using EMSA or SPR. EMSA was performed as follows (Berry et al., 2013; Cehovin et al., 2013). Biotin-labeled and non-labeled ds primers corresponding to DUS_{var1} or scrambled sequences (Table S2) were prepared by mixing equimolar amounts of two complementary oligonucleotides in 50 mM Tris (pH 8) and 100 mM NaCl, heating for 5 min at 95°C, and leaving to cool overnight. Purified MBP-$ComP_{sub}$ was prepared in the same buffer. An MBP-$ComP_{sub}$/DUS_{var1} complex was generated by mixing 5 fmol of biotinylated DUS_{var1} ds primer and 0.8 μM MBP-$ComP_{sub}$ (except for a DNA-only control) in 20 μl of 20 mM Tris-Cl (pH 8), 50 mM NaCl, and 2.5 mM Mg^{2+}, and incubating for 20 min at ambient temperature. Increasing concentrations of unlabeled competitor DNA (0.7, 2.8, 11.25, 45, and 180 pmol) were then added to the DNA-binding reactions, which were further incubated for 20 min at ambient temperature before being analyzed by native gel electrophoresis as described previously (Cehovin et al., 2013).

SPR was performed as follows (Berry et al., 2013; Cehovin et al., 2013). Equivalent amounts of biotin-labeled ds primers, prepared as above in 20 mM Tris (pH 8) and 150 mM NaCl, were coupled to neutravidin on the surface of a ProteOn NLC sensor chip resulting in ~265 RU as assessed on a ProteOn XPR36 protein interaction array system instrument (Bio-Rad). SPR was then performed by passing 10, 25, 50, 100, and 200 μM His_6-$ComP_{sub}$ (in 20 mM Tris [pH 8], 150 mM NaCl, 0.05% Tween 20) across the six available analyte channels of the chip at 60 μl min^{-1}, and the responses at equilibrium (R_{eq}) were recorded. A control trace was also collected using an empty ligand channel and used to normalize for non-specific binding effects. Four independent analyte injections were performed, with a regeneration step performed between each using 0.5 M NaCl. All experiments were carried out at 25°C.

ACCESSION NUMBERS

The X-ray crystal structure of ComP and NMR structure of $ComP_{sub}$ have been deposited in the PDB under accession codes PDB: 5HZ7 and 2NBA, respectively.

SUPPLEMENTAL INFORMATION

Supplemental Information includes three figures and two tables and can be found with this article online at http://dx.doi.org/10.1016/j.str.2016.04.001.

AUTHOR CONTRIBUTIONS

S.J.M. and V.P. designed and directed the research. All the experiments were done by J.L.B. Y.X. helped with NMR. P.N.W. and S.M.L. helped with SPR. J.L.B., S.J.M., and V.P. wrote the paper.

ACKNOWLEDGMENTS

This work was funded by the Biotechnology and Biological Sciences Research Council (BBSRC) and supported by the Wellcome Trust (via a Senior Investigator Award and a multi-user equipment grant to S.J.M.). We are grateful to Vivianne Goosens and Sophie Helaine (both from Imperial College London) for critical reading of the manuscript. We are grateful to Nathan J. Weyand (University of Arizona) for the gift of *N. subflava* genomic DNA.

Received: February 3, 2016
Revised: March 19, 2016
Accepted: April 4, 2016
Published: May 5, 2016

REFERENCES

Aas, F.E., Lovold, C., and Koomey, M. (2002). An inhibitor of DNA binding and uptake events dictates the proficiency of genetic transformation in *Neisseria gonorrhoeae*: mechanism of action and links to type IV pilus expression. Mol. Microbiol. *46*, 1441–1450.

Adams, P.D., Afonine, P.V., Bunkoczi, G., Chen, V.B., Davis, I.W., Echols, N., Headd, J.J., Hung, L.W., Kapral, G.J., Grosse-Kunstleve, R.W., et al. (2010). PHENIX: a comprehensive Python-based system for macromolecular structure solution. Acta Crystallogr. D Biol. Crystallogr. *66*, 213–221.

Ambur, O.H., Frye, S.A., and Tønjum, T. (2007). New functional identity for the DNA uptake sequence in transformation and its presence in transcriptional terminators. J. Bacteriol. *189*, 2077–2085.

Berry, J.L., and Pelicic, V. (2015). Exceptionally widespread nano-machines composed of type IV pilins: the prokaryotic Swiss Army knives. FEMS Microbiol. Rev. *39*, 134–154.

Berry, J.L., Cehovin, A., McDowell, M.A., Lea, S.M., and Pelicic, V. (2013). Functional analysis of the interdependence between DNA uptake sequence and its cognate ComP receptor during natural transformation in *Neisseria* species. PLoS Genet. *9*, e1004014.

Brown, D., Helaine, S., Carbonnelle, E., and Pelicic, V. (2010). Systematic functional analysis reveals that a set of 7 genes is involved in fine tuning of the multiple functions mediated by type IV pili in *Neisseria meningitidis*. Infect. Immun. *78*, 3053–3063.

Cehovin, A., Simpson, P.J., McDowell, M.A., Brown, D.R., Noschese, R., Pallett, M., Brady, J., Baldwin, G.S., Lea, S.M., Matthews, S.J., et al. (2013). Specific DNA recognition mediated by a type IV pilin. Proc. Natl. Acad. Sci. USA *110*, 3065–3070.

Chen, I., and Dubnau, D. (2004). DNA uptake during bacterial transformation. Nat. Rev. Microbiol. *2*, 241–249.

Chen, I., and Gotschlich, E.C. (2001). ComE, a competence protein from *Neisseria gonorrhoeae* with DNA-binding activity. J. Bacteriol. *183*, 3160–3168.

Cowtan, K. (2006). The Buccaneer software for automated model building. 1. Tracing protein chains. Acta Crystallogr. D Biol. Crystallogr. *62*, 1002–1011.

Craig, L., Volkmann, N., Arvai, A.S., Pique, M.E., Yeager, M., Egelman, E.H., and Tainer, J.A. (2006). Type IV pilus structure by cryo-electron microscopy and crystallography: implications for pilus assembly and functions. Mol. Cell *23*, 651–662.

Danner, D.B., Deich, R.A., Sisco, K.L., and Smith, H.O. (1980). An eleven-base-pair sequence determines the specificity of DNA uptake in *Haemophilus* transformation. Gene *11*, 311–318.

de Vries, S.J., van Dijk, A.D., Krzeminski, M., van Dijk, M., Thureau, A., Hsu, V., Wassenaar, T., and Bonvin, A.M. (2007). HADDOCK versus HADDOCK: new features and performance of HADDOCK2.0 on the CAPRI targets. Proteins *69*, 726–733.

Delaglio, F., Grzesiek, S., Vuister, G.W., Zhu, G., Pfeifer, J., and Bax, A. (1995). NMRPipe: a multidimensional spectral processing system based on UNIX pipes. J. Biomol. NMR *6*, 277–293.

Dominguez, C., Boelens, R., and Bonvin, A.M. (2003). HADDOCK: a protein-protein docking approach based on biochemical or biophysical information. J. Am. Chem. Soc. *125*, 1731–1737.

Emsley, P., Lohkamp, B., Scott, W.G., and Cowtan, K. (2010). Features and development of Coot. Acta Crystallogr. D Biol. Crystallogr. *66*, 486–501.

Frye, S.A., Nilsen, M., Tønjum, T., and Ambur, O.H. (2013). Dialects of the DNA uptake sequence in *Neisseriaceae*. PLoS Genet. *9*, e1003458.

Giltner, C.L., Nguyen, Y., and Burrows, L.L. (2012). Type IV pilin proteins: versatile molecular modules. Microbiol. Mol. Biol. Rev. *76*, 740–772.

Goodman, S.D., and Scocca, J.J. (1988). Identification and arrangement of the DNA sequence recognized in specific transformation of *Neisseria gonorrhoeae*. Proc. Natl. Acad. Sci. USA *85*, 6982–6986.

Griffith, F. (1928). The significance of pneumococcal types. J. Hyg. (Lon) *27*, 113–159.

Helaine, S., Dyer, D.H., Nassif, X., Pelicic, V., and Forest, K.T. (2007). 3D structure/function analysis of PilX reveals how minor pilins can modulate the virulence properties of type IV pili. Proc. Natl. Acad. Sci. USA *104*, 15888–15893.

Johnson, B.A., and Blevins, R.A. (1994). NMR View: a computer program for the visualization and analysis of NMR data. J. Biomol. NMR *4*, 603–614.

Luscombe, N.M., Austin, S.E., Berman, H.M., and Thornton, J.M. (2000). An overview of the structures of protein-DNA complexes. Genome Biol. *1*, REVIEWS001.

Maier, B., Chen, I., Dubnau, D., and Sheetz, M.P. (2004). DNA transport into *Bacillus subtilis* requires proton motive force to generate large molecular forces. Nat. Struct. Mol. Biol. *11*, 643–649.

Marchant, J., Sawmynaden, K., Saouros, S., Simpson, P., and Matthews, S. (2008). Complete resonance assignment of the first and second apple domains of MIC4 from *Toxoplasma gondii*, using a new NMRView-based assignment aid. Biomol. NMR Assign. *2*, 119–121.

Marri, P.R., Paniscus, M., Weyand, N.J., Rendon, M.A., Calton, C.M., Hernandez, D.R., Higashi, D.L., Sodergren, E., Weinstock, G.M., Rounsley, S.D., et al. (2010). Genome sequencing reveals widespread virulence gene exchange among human *Neisseria* species. PLoS One *5*, e11835.

McCoy, A.J., Grosse-Kunstleve, R.W., Adams, P.D., Winn, M.D., Storoni, L.C., and Read, R.J. (2007). Phaser crystallographic software. J. Appl. Crystallogr. *40*, 658–674.

Moon, A.F., Mueller, G.A., Zhong, X., and Pedersen, L.C. (2010). A synergistic approach to protein crystallization: combination of a fixed-arm carrier with surface entropy reduction. Protein Sci. *19*, 901–913.

Nivaskumar, M., Bouvier, G., Campos, M., Nadeau, N., Yu, X., Egelman, E.H., Nilges, M., and Francetic, O. (2014). Distinct docking and stabilization steps of the pseudopilus conformational transition path suggest rotational assembly of type IV pilus-like fibers. Structure *22*, 685–696.

Parge, H.E., Forest, K.T., Hickey, M.J., Christensen, D.A., Getzoff, E.D., and Tainer, J.A. (1995). Structure of the fibre-forming protein pilin at 2.6 Å resolution. Nature *378*, 32–38.

Provvedi, R., and Dubnau, D. (1999). ComEA is a DNA receptor for transformation of competent *Bacillus subtilis*. Mol. Microbiol. *31*, 271–280.

Rieping, W., Habeck, M., Bardiaux, B., Bernard, A., Malliavin, T.E., and Nilges, M. (2007). ARIA2: automated NOE assignment and data integration in NMR structure calculation. Bioinformatics *23*, 381–382.

Rusniok, C., Vallenet, D., Floquet, S., Ewles, H., Mouzé-Soulama, C., Brown, D., Lajus, A., Buchrieser, C., Médigue, C., Glaser, P., et al. (2009). NeMeSys: a resource for narrowing the gap between sequence and function in the human pathogen *Neisseria meningitidis*. Genome Biol. *10*, R110.

Seitz, P., Pezeshgi Modarres, H., Borgeaud, S., Bulushev, R.D., Steinbock, L.J., Radenovic, A., Dal Peraro, M., and Blokesch, M. (2014). ComEA is essential for the transfer of external DNA into the periplasm in naturally transformable *Vibrio cholerae* cells. PLoS Genet. *10*, e1004066.

Shen, Y., Delaglio, F., Cornilescu, G., and Bax, A. (2009). TALOS+: a hybrid method for predicting protein backbone torsion angles from NMR chemical shifts. J. Biomol. NMR *44*, 213–223.

Szabó, Z., Stahl, A.O., Albers, S.V., Kissinger, J.C., Driessen, A.J., and Pohlschröder, M. (2007). Identification of diverse archaeal proteins with class III signal peptides cleaved by distinct archaeal prepilin peptidases. J. Bacteriol. *189*, 772–778.

Thomas, C.M., and Nielsen, K.M. (2005). Mechanisms of, and barriers to, horizontal gene transfer between bacteria. Nat. Rev. Microbiol. *3*, 711–721.

Webb, B., and Sali, A. (2014). Comparative protein structure modeling using MODELLER. Curr. Protoc. Bioinformatics *47*, 5.6.1–5.6.32.

Winter, G. (2010). xia2: an expert system for macromolecular crystallography data reduction. J. Appl. Crystallogr. *43*, 186–190.

Wolfgang, M., Lauer, P., Park, H.S., Brossay, L., Hébert, J., and Koomey, M. (1998). PilT mutations lead to simultaneous defects in competence for natural transformation and twitching motility in piliated *Neisseria gonorrhoeae*. Mol. Microbiol. *29*, 321–330.

Wolfgang, M., van Putten, J.P., Hayes, S.F., and Koomey, M. (1999). The *comP* locus of *Neisseria gonorrhoeae* encodes a type IV prepilin that is dispensable for pilus biogenesis but essential for natural transformation. Mol. Microbiol. *31*, 1345–1357.

Structure, Volume *24*

Supplemental Information

A Comparative Structure/Function Analysis of Two Type IV Pilin DNA Receptors Defines a Novel Mode of DNA Binding

Jamie-Lee Berry, Yingqi Xu, Philip N. Ward, Susan M. Lea, Stephen J. Matthews, and Vladimir Pelicic

A comparative structure/function analysis of two type IV pilin DNA receptors defines a novel mode of DNA-binding

Jamie-Lee Berry[1], Yingqi Xu[2], Philip N. Ward[3], Susan M. Lea[3], Stephen J. Matthews[2,*] and Vladimir Pelicic[1,*]

[1]MRC Centre for Molecular Bacteriology and Infection, Imperial College London, London SW7 2AZ, United Kingdom

[2]Centre for Structural Biology, Imperial College London, London SW7 2AZ, United Kingdom

[3]Sir William Dunn School of Pathology, University of Oxford, Oxford OX1 3RE, United Kingdom

*Correspondence: s.j.matthews@imperial.ac.uk (S.J.M.), v.pelicic@imperial.ac.uk (V.P.)

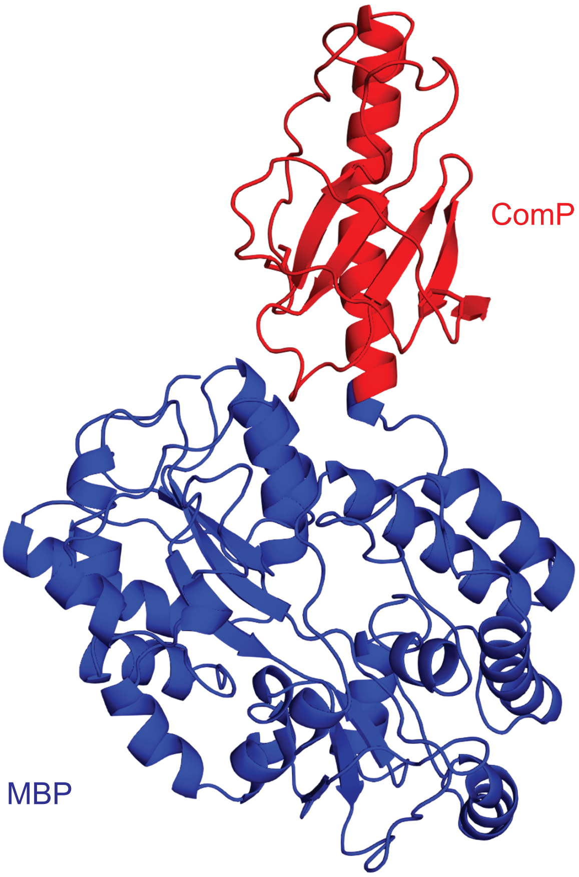

Figure S1, related to Figure 3. Full 3D structure of the MBP-ComP fusion protein. The crystal structure is shown as a cartoon drawing. The ComP moiety is highlighted in red, while the MBP moiety is highlighted in blue.

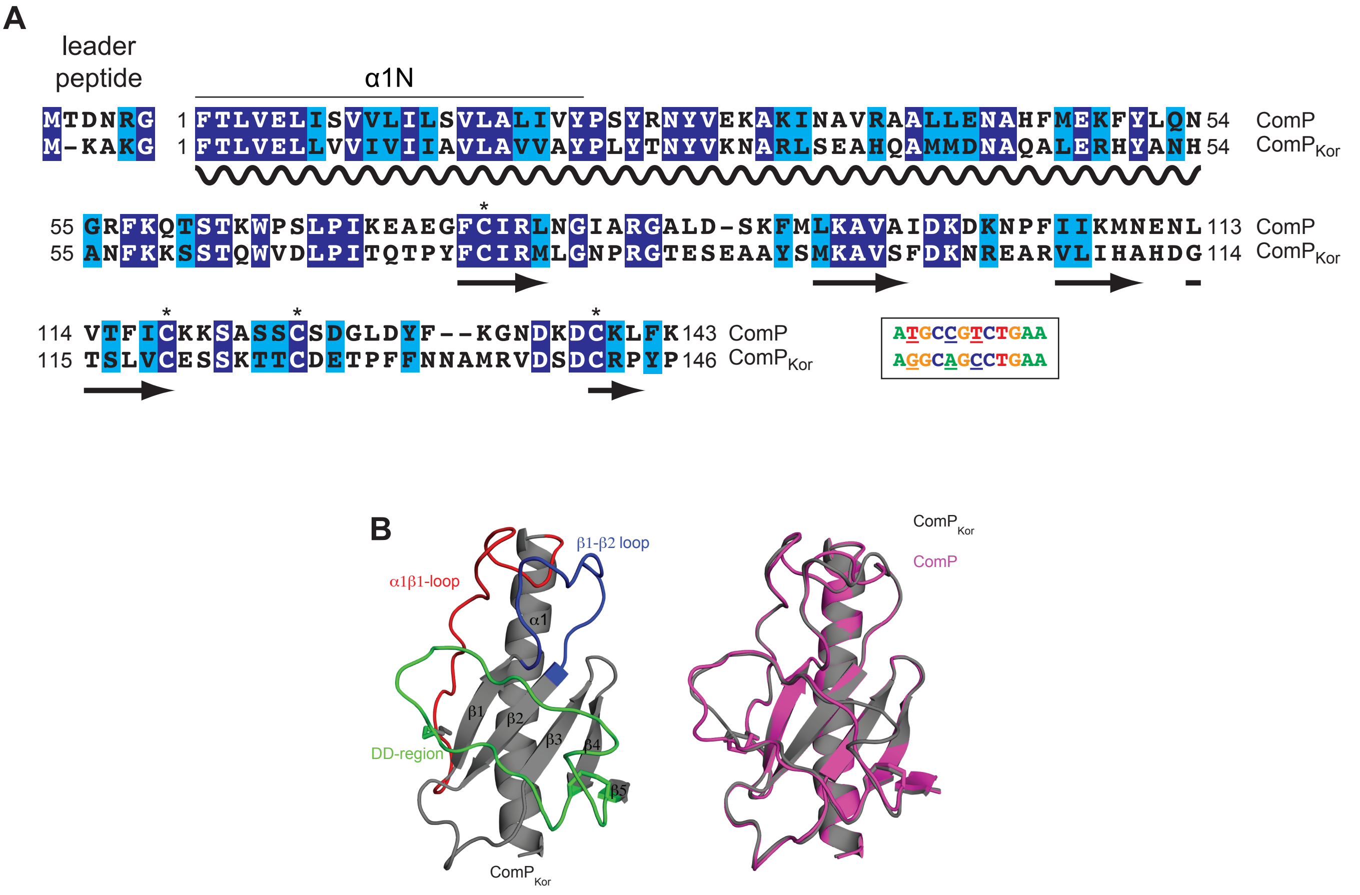

Figure S2, related to Figures 3 and 4. Homology modelling of the structure of the ComP$_{Kor}$ ortholog from *K. oralis*. (A) Sequence alignment of ComP$_{Kor}$ with ComP from *N. meningitidis* 8013, produced using Clustal Omega. Amino acids were shaded in dark blue (when identical), in light blue (when highly similar) or non-shaded (when non-conserved). Relevant structural and functional features have been highlighted. The four Cys residues that form two crucial disulfide bonds are identified by *. The inset represents a sequence alignment of DUS and the DUS variant found in *K. oralis*. These motifs differ by three base, which have been underlined. (B) Cartoon drawing representation of the homology model of ComP$_{Kor}$ and of its superposition with the ComP crystal structure.

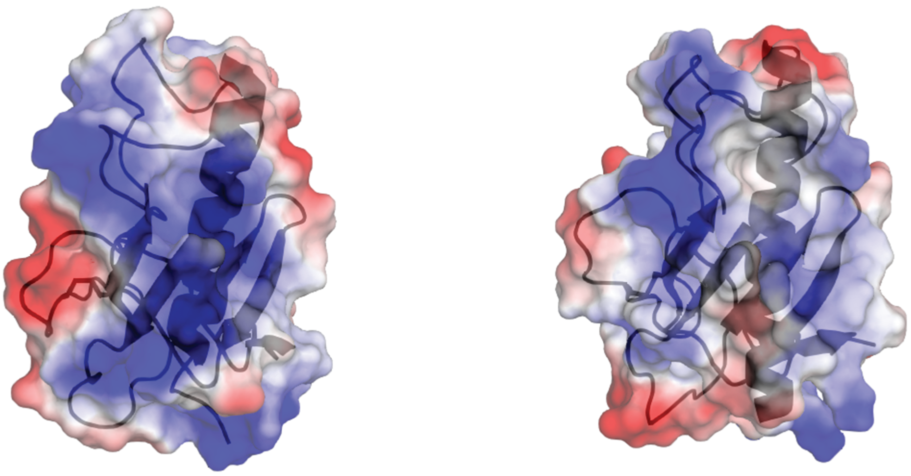

ComP $\qquad$ $ComP_{sub}$

Figure S3, related to Figures 3 and 4. Surface charge representation of the ComP and $ComP_{sub}$ structures. Positively charged residues are represented in blue, negatively charged residues in red, while neutral residues are not coloured.

Table S1, related to Figures 3 and 4. Structural data collection and refinement parameters.

**MBP-ComP**	
Resolution range (Å)	58.05-1.43 (1.47-1.43)
Space group	$P2_12_12_1$
Unit cell dimensions	63.57, 68.44, 109.58; 90, 90, 90
Total observations	415,797 (28,880)
Total unique	88,446 (6,445)
Multiplicity	4.7 (4.5)
Completeness (%)	99.5 (99.2)
I/σ(I)	15 (1.2)
Wilson B factor	21.3
R_{merge}	0.039 (1.096)
R_{meas}	0.049 (1.384)
R_{work}	0.16
R_{free}	0.19
Ramachandran favoured (%)	97.2
Ramachandran outliers (%)	0
Average B factor	29
**His_6-$ComP_{sub}$**	
Number of distance restraints	2,017
intra-residual	744
sequential	445
medium range	273
long range	555
NOE violations >0.5 Å	1
Dihedral violations >5º	0
Ramachandran favoured (%)	75.5
Ramachandran allowed (%)	20.6
Ramachandran generously allowed (%)	3.9
Ramachandran disallowed (%)	0

Table S2, related to the Experimental proocedures. Primers used in this study.

Name	Sequence[a]
**Cloning**	
opt*comP*-F	gg**gaattc**GAAAAAGCCAAAATTAACGCAGTT
opt*comP*-R	gg**aagctt**TTATTTAAACAGTTTGCAGTCTTTG
comP$_{sub}$-pMalF	gg**gaattc**CGCTCGGCCAACCTGCGTG
comP$_{sub}$-pMalR	gg**aagctt**TCACCCCGTAAAAGGCCGA
his*comP*$_{sub}$-pETF	gg**ccatgg**atcatcatcatcatcatcatCGCTCGGCCAACCTGCGTG
his*comP*$_{sub}$-pETR	cc**ggatcc**TCACCCCGTAAAAGGCCGA
**DNA-binding assay (EMSA/SPR)**	
DUS$_{var1}$1[b]	tgaccAGGCCGTCTGAAcaaac
DUS$_{var1}$2	gtttgTTCAGACGGCCTggtca
SUD1[b]	tgaccACGACTTATAATcaaac
SUD2	gtttgATTATAAGTCGTggtca
SDU1[b]	tgaccAAGGCCTGTCATcaaac
SDU2	gtttgATGACAGGCCTTggtca
**NMR titration**	
DUS$_{var1}$1bis	cAGGCCGTCTGAAc
DUS$_{var1}$2bis	gTTCAGACGGCCTg

[a]Overhangs are in lower case, with restriction sites in bold.

[b]When indicated, these primers were 5'-labelled with biotin.
